# Telomerase Activity Impacts on Epstein-Barr Virus Infection of AGS Cells

**DOI:** 10.1371/journal.pone.0123645

**Published:** 2015-04-09

**Authors:** Jürgen Rac, Florian Haas, Andrina Schumacher, Jaap M. Middeldorp, Henri-Jacques Delecluse, Roberto F. Speck, Michele Bernasconi, David Nadal

**Affiliations:** 1 Experimental Infectious Diseases and Cancer Research, University Children’s Hospital of Zurich, University of Zurich, Zurich, Switzerland; 2 Division of Infectious Diseases and Hospital Epidemiology, University Children’s Hospital of Zurich, University of Zurich, Zurich, Switzerland; 3 Children’s Research Center, University Children’s Hospital Zurich, University of Zurich, Zurich, Switzerland; 4 Department of Pathology and Cancer Center Amsterdam, Vrije Universiteit Medical Center, Amsterdam, The Netherlands; 5 Division of Pathogenesis of Virus Associated Tumors, German Cancer Research Center, Heidelberg, Germany; 6 Division of Infectious Diseases and Hospital Epidemiology, Department of Medicine, University Hospital Zurich, University of Zurich, Zurich, Switzerland; Georgetown University, UNITED STATES

## Abstract

The Epstein-Barr virus (EBV) is transmitted from host-to-host via saliva and is associated with epithelial malignancies including nasopharyngeal carcinoma (NPC) and some forms of gastric carcinoma (GC). Nevertheless, EBV does not transform epithelial cells *in vitro* where it is rapidly lost from infected primary epithelial cells or epithelial tumor cells. Long-term infection by EBV, however, can be established in hTERT-immortalized nasopharyngeal epithelial cells. Here, we hypothesized that increased telomerase activity in epithelial cells enhances their susceptibility to infection by EBV. Using HONE-1, AGS and HEK293 cells we generated epithelial model cell lines with increased or suppressed telomerase activity by stable ectopic expression of hTERT or of a catalytically inactive, dominant negative hTERT mutant. Infection experiments with recombinant prototypic EBV (rB95.8), recombinant NPC EBV (rM81) with increased epithelial cell tropism compared to B95.8, or recombinant B95.8 EBV with *BZLF1*-knockout that is not able to undergo lytic replication, revealed that infection frequencies positively correlate with telomerase activity in AGS cells but also partly depend on the cellular background. AGS cells with increased telomerase activity showed increased expression mainly of latent EBV genes, suggesting that increased telomerase activity directly acts on the EBV infection of epithelial cells by facilitating latent EBV gene expression early upon virus inoculation. Thus, our results indicate that infection of epithelial cells by EBV is a very selective process involving, among others, telomerase activity and cellular background to allow for optimized host-to-host transmission via saliva.

## Introduction

Epstein-Barr virus (EBV) is transmitted *via* saliva and has to pass the oral mucosal epithelium after exiting from B cells, the site where the virus establishes latency. The source of EBV infectious progeny in saliva remains elusive [1–3]. It has been demonstrated that differentiation of memory B cells into plasma cells results in reactivation of latent EBV and virus replication [4]. Nevertheless, EBV is believed to reside and replicate also in oropharyngeal epithelium [5,6]. Notably, cell-free EBV predominantly infects epithelial cells from the basolateral membranes [7], and cell-associated virus efficiently infects cells from the apical surface [8] especially after cell-to-cell contact [9]. Recent work has shown that cell-associated EBV infects *in vitro* reconstituted stratified epithelium from its mucosal surface [10]. Since EBV egressing from epithelial cells is more lymphotropic than EBV egressing from B cells [11], lytic replication in oropharyngeal epithelial cells might be important for efficient host-to-host transmission.

The oral mucosal epithelium is a dynamic tissue with a distinct multilayer architecture [12]. Its basement membrane separates the epithelium from the underlying *lamina propria* and ensures correct and directed migration and differentiation of the overlying epithelial cells towards the surface of the epithelium. The *stratum basale*, a single layer of cells resting on the basement membrane, is most important for tissue hemostasis. The *stratum basale* harbors a small sub-population of epithelial stem cells, which can undergo mitotic division and give rise to transiently proliferating progenitor cells [12,13]. The transiently proliferating cells then can generate daughter cells that migrate and differentiate through the *stratum spinosum* and *stratum granulosum* towards the epithelial surface, the *stratum corneum*. Epithelial stem cells have an increased expression and activity of the human telomerase reverse transcriptase (hTERT), the rate-limiting component of the telomerase complex, to ensure indefinite proliferation and continuous self-renewal capacity [13–17]. Since epithelial cells differ considerably depending on their site of origin and differentiation stage and exhibit variable binding of EBV [7], the stable infection of epithelial cells by EBV is likely to be a very selective process, linked among others to the cell differentiation state.

EBV is associated with epithelial cell carcinomas including nasopharyngeal carcinoma (NPC) and gastric carcinoma (GC) where the virus expresses latency genes [18]. *In vitro*, EBV is rapidly lost from infected primary epithelial cells or from epithelial tumor cells [19–22]. Nonetheless, it is possible to establish hTERT-immortalized nasopharyngeal epithelial cell clones that are able to support a long-term infection by EBV. It appears that loss or inactivation of the tumor suppressor p16 and cyclin D1 overexpression are crucial events for the establishment and the support of a stable EBV infection [23–25]. Both events are common in NPC and GC development [26–30] and impact on telomerase activity [31]. Thus, cells with enhanced survival potential seem to be more susceptible to EBV infection.

On the other hand, EBV itself has the ability to induce telomerase activity in B-cells [32–34] through LMP1, the major EBV-encoded oncogene. Notably, LMP1 induces telomerase activity *via* NF-κB activation in B cells and after ectopic expression in epithelial cells [35–37]. Furthermore, LMP2A affects hedgehog signaling and induces stem cell behavior in epithelial cells [38] and BARF1 may trigger expression of cyclin D1 in epithelial cells [39]. Therefore, upon entry into epithelial cells and following expression of its main latency gene products, EBV may create conditions for its own persistence and alter epithelial cell functions, provided that appropriate signaling adapter molecules are present in the infected cell. This may be different in epithelial cells from different origin and has received little attention thus far. Importantly, hTERT contributes to EBV maintenance by induction of EBV latent gene expression and down-regulation of lytic EBV gene expression in early-passage infected B lymphocytes [40]. Moreover, hTERT inhibition might promote lytic EBV replication in EBV-immortalized and fully transformed B cells [41], thus providing a potential therapeutic target. Nevertheless, the impact of hTERT expression and telomerase activity on EBV infection in epithelial cells remains to be elucidated.

Here, we hypothesized that increased telomerase activity in epithelial cells can enhance their susceptibility to infection by EBV. Thus, we generated epithelial model cell lines (i) with increased telomerase activity, by ectopic expression of hTERT, and (ii) with lowered telomerase activity, by ectopic expression of a catalytically inactive DNhTERT. Subsequently, we assessed the EBV infection frequencies and virus transcriptional activity in the model cell lines after inoculation with three EBV strains: (i) the reference strain B95.8, (ii) M81 with increased tropism for epithelial cells, and (iii) B95.8 with *BZLF1* knockout that is impaired for lytic replication.

## Material and Methods

### Cells and Viruses

As epithelial model cell lines we used the nasopharyngeal carcinoma (NPC) cell line HONE-1 [20], maintained in RPMI-1640 (Sigma-Aldrich, Buchs, Switzerland), the gastric carcinoma cell line AGS [42], maintained in HAM’s F-12 (Sigma-Aldrich) and the human embryonic kidney cell line HEK293 [43], maintained in Dulbecco’s Modified Eagle’s Medium (DMEM; Sigma-Aldrich). All media were supplemented with 10% heat inactivated Fetal Bovine Serum (hiFBS; Sigma-Aldrich), 1% L-Glutamine and 1% Penicillin/Streptomycin (Gibco, Zug, Switzerland).

Supernatant containing the recombinant EBV strain rM81 with more pronounced epithelial cell tropism [44] was kindly provided by Prof. H.-J. Delecluse (DKFZ Heidelberg, Germany). The EBV producer cell lines HEK293-rB95.8 [45], for the production of the prototypic EBV strain B95.8 (rB95.8), and HEK293-rBZLF1-KO [46], for the production B95.8 virus with a *BZLF1*-knockout (rBZLF1-KO) that is therefore not capable of lytic replication, were maintained in DMEM (Sigma-Aldrich) supplemented with 10% hiFBS, 1% L-Glutamine, 1% Penicillin/Streptomycin, 100 μg/ml Hygromycin B (HygroGOLD; InvivoGen, Toulouse, France). Virus-containing supernatants were produced as described elsewhere [47]. Briefly, 80–90% confluent HEK293-rB95.8 cells were transfected with expression plasmids encoding the EBV gene *BZLF1*, to induce lytic replication, and *BALF4*, to optimize gp110 levels on the viral surface [48], (2 μg each/10 cm plate) using Metafectene (Biontex, Martinsried/Planegg, Germany). Four hours after transfection, the transfection mixture was replaced by fresh supplemented DMEM without Hygromycin B. Three to four days after transfection, supernatants were harvested, cleared by centrifugation at 4°C with 1.000 × g for 15 min, filtered through a 0.45 μm filter and stored at -80°C. Concentrated virus stocks were prepared by centrifugation of viral supernatant with 30.000 × g for 2.5 h at 4°C and resuspension of the virus pellet in 1x PBS (1/100 of the starting Volume). The number of infectious EBV units was determined as described elsewhere and virus titers are given as multiplicity of infection (MOI) and defined as infectious units/target cell [47,49]. For the virus strains rM81 and rBZLF1-KO we additionally determined the amount of EBV genome equivalents/ml supernatant or concentrated virus by quantitative PCR using a *LMP1*-specific primer/probe set as described previously [50]. Virus supernatants or concentrated virus stock were subjected to DNase treatment using the Ambion DNAfree kit (Applied Biosystems, Zug, Switzerland) before DNA isolation to estimate the amount of encapsidated viral genomes and therefore potentially infectious virus. The amount of virus, used for the infections, was adjusted for each virus strain corresponding to the highest MOI of 2.5 infectious units/target cell.

### Generation of hTERT and DNhTERT overexpressing epithelial cell lines

To generate hTERT overexpressing cells we employed the expression vector pWZL-Blast-Flag-HA-hTERT [51], kindly provided by William C. Hahn (Harvard Medical School, Cambridge, MA, USA). To generate cells expressing the dominant negative hTERT mutant (DNhTERT) we exchanged the hTERT insert from pWZL-Blast-Flag-HA-hTERT with the DNhTERT mutant from the expression vector pBABE-DNhTERT [52], kindly provided by Robert Weinberg (Whitehead Institute for Biomedical Research, Cambridge, MA, USA), using the *Eco*RI/*Sal*I restriction sites. Empty control vector was generated by excision of the hTERT insert from pWZL-Blast-Flag-HA-hTERT, using the *Xho*I/*Sal*I restriction sites and subsequent re-ligation. We then transfected either 10^6^ HONE-1, AGS or HEK293 cells with the hTERT-, DNhTERT or the empty vector (later referred as HONE-1, AGS or HEK293-EV,-hT and-DN), respectively, using Metafectene and 2 days post transfection we selected for resistant cells and maintained the cells with the addition of 10 μg/ml Blasticidin (InvivoGen) to the normal growth medium to establish stable cell lines.

### Gene expression analysis by RT-qPCR

Gene expression was determined by quantitative reverse transcription polymerase chain reaction (RT-qPCR) using specific primers and probes for the housekeeping gene *HMBS*, the non-coding EBV encoded RNA *EBER1*, the latency associated EBV genes *EBNA1*, *EBNA2*, *LMP1* and *LMP2A* and for the two genes related to the lytic replication cycle of EBV, *BZLF1* and *BXLF2*, as described earlier [53–55]. Additionally, to detect *BZLF1* gene expression in rM81 infected cells we used a different forward primer for the *BZLF1* (5’-CAC GAC GTA CAA GGA AAC-3’) and *LMP1* (5’-TGG AGG CCT TGG TCT ACT CCT-3’) primer/probe set, which we termed *aBZLF1* and *aLMP1*, respectively, due to the sequence homology to the Akata EBV strain. Gene expression of *BARF1* was determined using the forward primer 5’-GAG CCT CTC TGT TGC TGT TG-3’, the probe 5’-FAM-TCC CAA CGC AGG TCA CTG GC-BHQ1-3’ and the reverse primer 5’-GGG CTT CCT CCT TGT CAT T-3’. Gene expression of *hTERT* and *DNhTERT* was determined using a pre-validated primer/probe assay (Hs00972656; Applied Biosystems). Therefore, total RNA was isolated at the indicated hours or days, respectively, post inoculation (hpi and dpi, respectively) using the RNeasy Mini Kit (Qiagen, Hombrechtikon, Switzerland), followed by DNase treatment (DNA-free Kit; Ambion, Zug, Switzerland) and cDNA synthesis from 0.5 μg RNA using a High Capacity cDNA Reverse Transcription Kit (Applied Biosystems) according to manufacturers instructions. All reactions were performed in triplicates for each condition and gene on an ABI Prism 7700 real-time PCR machine (Applied Biosystems). Results were analyzed with the software SDSv2.3 (Applied Biosystems) and gene expression was calculated relative to the housekeeping gene *HMBS* using the 2^-dCt method. Cycle threshold (Ct) values from technical replicates with standard de*via*tions (SD) > 0.5 were excluded from gene expression calculations. Cts above 36, resulting in relative gene expression levels below 0.001, defined the limit of detection since most of these values became unreliable above this threshold regarding their SD.

### Western Blot analysis

To determine Telomerase protein levels by western blot, whole-cell extracts were prepared from 10^6^ cells using RIPA buffer (50 mM Tris-Cl, pH 6.8, 100 mM NaCl, 1% Triton X-100, 0.1% sodium dodecyl sulfate) supplemented with complete mini protease inhibitor cocktail (Roche, Rotkreuz, Switzerland). After determination of the protein concentration using the Pierce BCA Protein Assay Kit (Thermo Scientific, Wohlen, Switzerland), protein extracts were separated on 4–12% NuPAGE Bis-Tris Precast gels (Invitrogen, Zug, Switzerland) and proteins were semi-dry transferred for 45 min with 25 V on nitrocellulose membranes (Optitran BA-S83; Whatman, Wohlen, Switzerland). hTERT and DNhTERT protein were probed with the primary Telomerase reverse Transcriptase antibody Y182 (1:500; Novus Biologicals, Luzern, Switzerland) and as loading control β-Actin was probed with the primary β-Actin antibody (dilution 1:5000; #4967, Cell Signaling, Allschwil, Switzerland). Primary antibodies were detected using a horseradish peroxidase-conjugated goat anti-rabbit IgG (dilution 1:5000; #7074, Cell Signaling). Signals were obtained by incubation with the SuperSignal West Femto Chemiluminescent Substrate (Thermo Scientific) following manufacturer instructions and visualized on the Image Reader LAS-3000 (Fujifilm, Tokyo, Japan).

### Telomeric repeat amplification protocol (TRAP) assay

Telomerase activity was determined using the TRAPeze Telomerase Detection Kit (S7700; Millipore, Zug, Switzerland) following manufacturers instructions with following modifications. Protein concentrations were determined using the Pierce BCA Protein Assay Kit (Thermo Scientific). The reactions were carried out with 1:50 diluted cell lysates, corresponding to 100 cells. Telomerase extension reaction was performed at 30°C for 30 min followed by 2 min denaturation at 94°C and addition of 2 units *Taq* Polymerase per reaction. Amplification of the telomeric repeats was done in 30 cycles including denaturation at 94°C for 5 s, annealing at 55°C for 30 s, elongation at 72°C for 1 min and a final single-step elongation at 72°C for 5 min. TRAP reactions were separated on 10% TBE gels (Invitrogen) and products were visualized after staining with SYBR Green I nucleic acid gel stain, according to manufacturers instructions (Sigma-Aldrich) using the GeneFlash gel documentation system (Syngene, Châtel-St-Denis, Switzerland).

### Direct inoculation of epithelial cells with cell-free virus by spinoculation

For the direct inoculation of epithelial cells with cell-free virus or concentrated virus we employed an adapted spinoculation protocol to achieve measurable frequencies of infection [54]. Briefly, 10^5^ cells were seeded in 12-well plates and incubated over night at 37°C with 5% CO_2_. Target cells were then inoculated by adding cell-free virus supernatant or concentrated virus with varying MOIs, as indicated, to the target cells in a total volume of 500 μl to ensure equal virus concentrations. Then cells were centrifuged for 1 h at 32°C with 800 × g, supernatant was aspirated, replaced by 1 ml fresh medium and incubated at 37°C with 5% CO_2_. To determine infection frequencies at the indicated hpi or dpi, cells were detached using 0.25% Trypsin-EDTA (Gibco), washed with 1x phosphate buffered saline (PBS; Gibco), stained with the cell *via*bility dye 7-Amino-Actinomycin D (7-AAD; BD Bioscience, Allschwil, Switzerland), to exclude non-*via*ble cells, according to manufacturer’s instructions, washed again with 1x PBS and the amount of GFP positive (GFP+; infected cells) was determined by flow cytometry using the FACS Canto II (BD Bioscience) within the living cell population. Mock inoculations of each cell line were performed without virus and the amount of false GFP+ cells, detected as background signals, were subtracted from corresponding inoculations.

### Detection of EBV infected cells by fluorescence *in situ* hybridization (FISH)

To validate and confirm infection of AGS cells, *EBER*-FISH was performed using DIG-labeled probes specific for *EBER*s (PanPath, Budel, Netherlands). Hybridization was performed according to the manufacturer’s instructions. Briefly, inoculated cells were seeded on microscopy chamber slides (BD Falcon CultureSlides, BD Bioscience). At the indicated time points cells were fixed with 4% Roti-Histofix (Carl Roth AG, Arlesheim, Switzerland) for 15 min at room temperature (RT), washed with 1x PBS, rinsed with ddH_2_O and dehydrated in 100% EtOH. Subsequently, cells were hybridized with the *EBER*-probes for 2 h at 37°C in a moist environment. Slides were then washed with 1x PBS and incubated with the secondary Dylight594-labeled anti-Digoxigenin antibody (Vector Laboratories, RECATOLAB, S.A., Servion, Switzerland) for 30 min at RT, washed again with 1x PBS and rinsed with ddH_2_O. After removing most of the remaining liquid, slides were mounted with VECTASHIELD Mounting Medium (Vector Laboratories) containing 4’,6-diamidino-2-phenylindole (DAPI) to counterstain the nucleus. Slides were analyzed using a fluorescence microscope Axioskop 2 MOT plus (Carl Zeiss, Jena, Germany) and images were process with the AxioVision Rel. 4.8.2 software (Carl Zeiss).

### Statistical analysis

Data sets were tested for statistical significance as indicated using Prism6 (GraphPad, La Jolla, CA, USA) and *P* values <0.05 were regarded as statistically significant.

## Results

### Generation of epithelial cell lines with increased telomerase expression or expression of the dominant negative telomerase mutant

**Fig 1:**
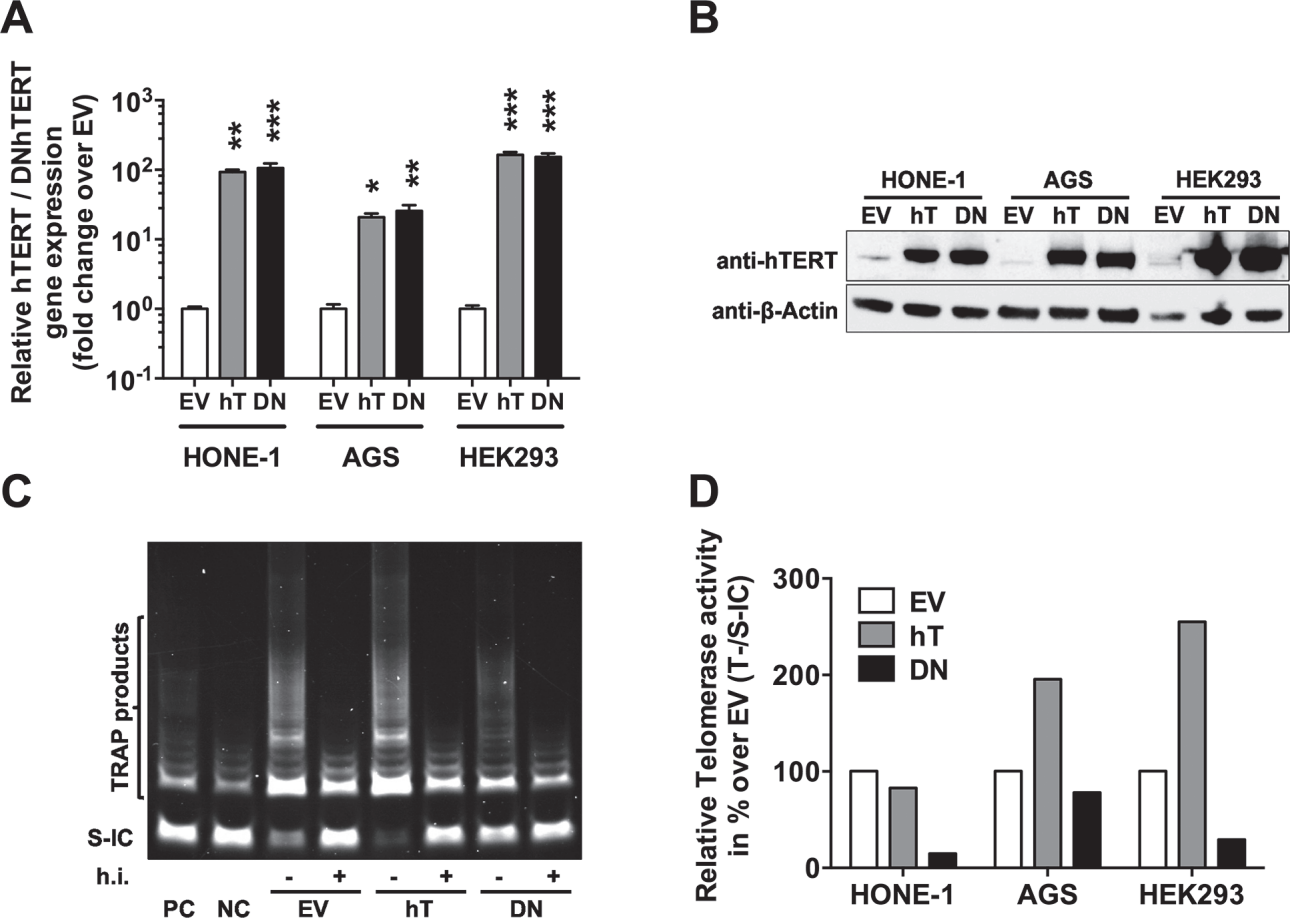
Increased hTERT/DNhTERT expression and altered telomerase activity in engineered epithelial cell lines. A) hTERT and DNhTERT mRNA levels were determined in empty vector control (EV; white), in hTERT- (hT; grey) and DNhTERT- (DN; black) overexpressing cells by RT-qPCR relative to *HMBS* and shown as fold change over EV. Data is shown as Mean ±SEM of 3 independent experiments. * = p<0.05; ** = p<0.01; *** = p<0.001 (ordinary one-way ANOVA; Dunnett’s multiple comparison test). B) Protein expression was confirmed by western blot using β-Actin as loading control. Telomerase activity (T) was determined by TRAP assay (C; representative assay) relative to corresponding standard internal controls (S-IC) in empty vector (EV) control, in hTERT- (hT) and DNhTERT- (DN) overexpressing cells and shown relative to corresponding EV control cells (D). Data is represented as Mean from triplicate measurements. PC = positive control; NC = negative control; h.i. = heat inactivated.

To address the question whether hTERT expression level influences EBV infection in epithelial cells, we set out to establish an *in vitro* model. For this we chose three EBV-negative epithelial cell lines: HONE-1, which originates from an EBV-positive NPC but lost EBV *in vitro* [20,21], suggesting that they do no longer support EBV infection; the gastric carcinoma cell line AGS, which is often used to study epithelial cell infection with EBV [5,11,19,56]; and HEK293, deriving from human epithelial kidney and thus anatomically remote from the oropharynx [43]. The cell lines were stably transfected either with hTERT (hT), the rate limiting component of the human telomerase complex, the telomerase reverse transcriptase, or its catalytically inactive mutant DNhTERT (DN) [52]. Empty vector (EV) transfected cells served as control. To confirm overexpression in hTERT- and DNhTERT-transfected cells, we investigated gene and protein expression (Fig 1A and 1B). Compared to EV control cells, we observed increased *hTERT* and *DNhTERT* expression in HONE-1 cells by 92.9 ± 13.1 and 106.0 ± 30.2 fold, respectively; in AGS cells 20.9 ± 4.7 and 25.7 ± 9.1 fold, respectively; and in HEK293 cells 162.7 ± 28.8 and 153.3 ± 31.8, respectively (Fig 1A). Western blot analysis (Fig 1B) confirmed increased protein expression of hTERT and DNhTERT in HONE-1, AGS, and HEK293 cells.

Next, we asked how overexpression of hTERT and DNhTERT impacts on telomerase activity by using the Telomeric Repeat Amplification Protocol (TRAP assay). The assay is a two step *in vitro* assay that mimics the *in vivo* telomerase function, elongation and maintenance of telomere length by synthesis and guidance of 6 base telomeric repeats (TTAGGG) to the 3’ ends of existing telomeres. In the first step, native, whole-cell protein extract, containing active telomerase, is utilized to add telomeric repeats to the 3’ ends of synthetic oligonucleotide substrates (TS). The second step is the amplification of these extended TRAP products by PCR, thus generating a ladder of fragments with 6 nucleotide increments, starting at 50 nucleotides, which then can be separated by polyacrylamide gel electrophoresis. Fig 1C shows a representative result for HEK293 cells. Endogenous telomerase activity was readily detected in all three EV control cell lines (Fig 1D). Telomerase activity in AGS-hT and HEK293-hT cells was increased to 195.6% and 255.1, respectively, compared to corresponding EV control cells (Fig 1D). In contrast, the ectopic expression of hTERT did not lead to an increase in telomerase activity in HONE-1-hT cells, instead we detected slightly decreased telomerase activity in HONE-1-hT cells (82.9%) compared to the EV controls (Fig 1D). This contrasted the robust increase observed at the gene and protein level (Fig 1A and 1B). The rather moderate change in telomerase activity in HONE-1-hT cells is in line with findings of Hahn and colleagues [52]. The telomerase activity in HONE-1-EV and HEK293-EV cells was 8.8-fold and 6.4-fold, respectively, whereas that of AGS-EV 1.7-fold in relation to the internal standard control as determined by TRAP assay. Thus, the relatively high telomerase activity in parental HONE-1 cells might have impeded additional increase of telomerase activity in HONE-1-hT cells.

The expression of DNhTERT led to suppression of telomerase activity below endogenous levels in all three cell lines with the strongest reduction of 85.1% observed in HONE-1-DN cells followed by reduction of 70.7% in HEK293-DN cells and of 21.9% in AGS-DN cells compared to in the respective EV control cells. Nevertheless, we did not observe any obvious sign of growth inhibition or senescence in DNhTERT-transfected cell lines as observed by Hahn *et al*. [52]. All cell lines stably expressing DNhTERT showed a growth behavior in culture similar to their corresponding controls and hTERT cells. Cell clones with sufficient amounts of DNhTERT to completely block telomerase activity might indeed stop proliferating, eventually becoming apoptotic [52], and be lost during the selection procedure. Indeed, we did observe single cells with the characteristic large and flattened, crisis-associated morphology in the stably DNhTERT-transfected cell lines, indicating that some cells stopped proliferating.

Taken together, although expression of hTERT protein was significantly increased in all three stably transfected cell lines, telomerase activity was markedly increased only in AGS-hT and HEK293-hT cells. The expression of DNhTERT led to consistent marked decrease of telomerase activity in all three stably transfected cell lines.

### GFP and RNA carryover can be distinguished from *de novo* expression

**Fig 2:**
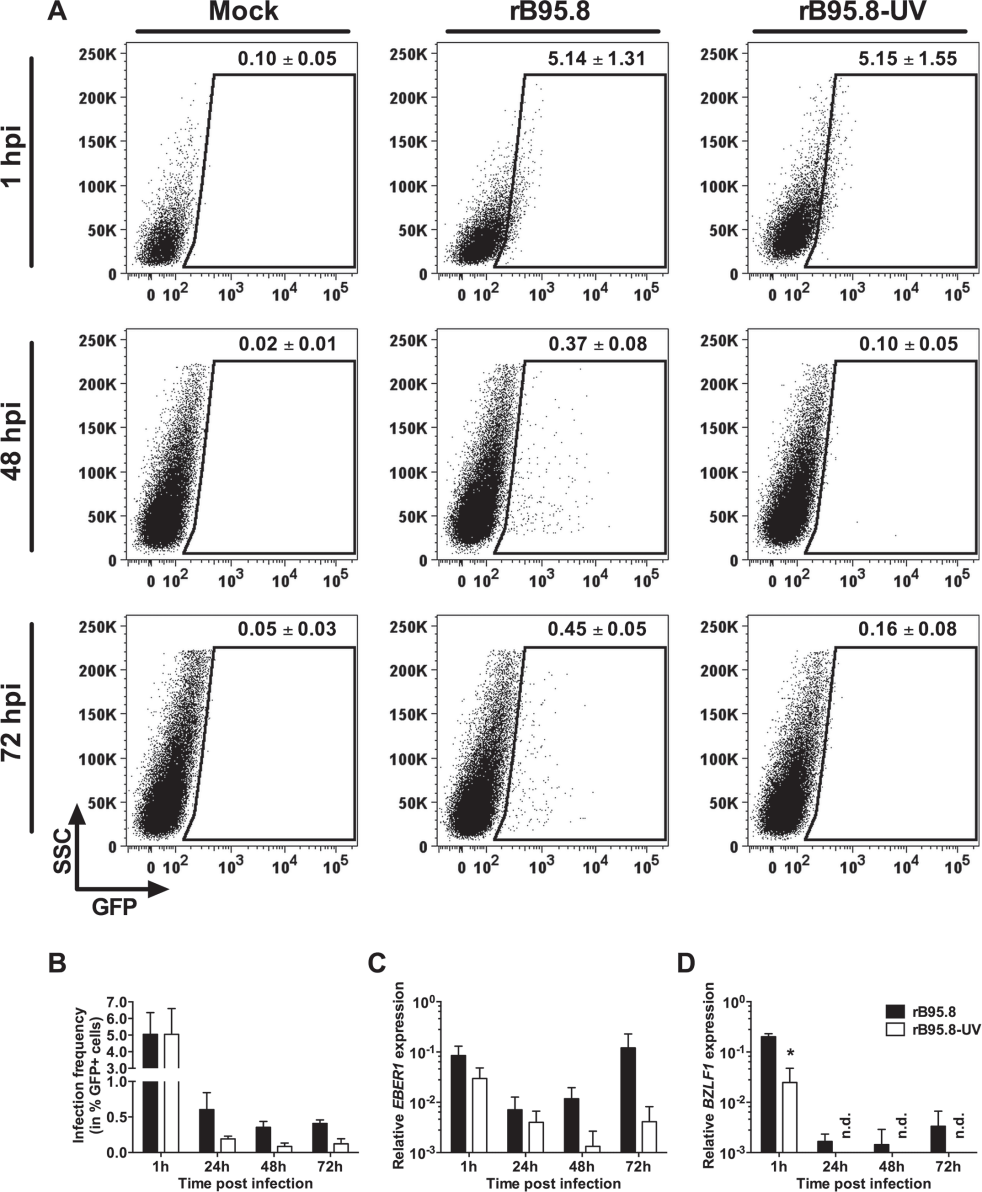
GFP and EBV gene expression early upon inoculation of HEK293 cells with rB95.8. A) HEK293 cells were either mock (without virus) inoculated or with rB95.8 and UV-inactivated rB95.8 (rB95.8-UV) at a MOI of 0.5 (infectious units/target cell), respectively. The amount of GFP+ cells (shown as Mean ± SEM of 3 independent inoculations above the SSC/GFP gate) was determined within the living cell (7-AAD negative) population by flow cytometry at 1 hour (upper panel) and 72 hours (lower panel) post infection (hpi). Dot plots show representative samples of triplicates from 3 independent inoculations. B) HEK293 cells were inoculated and analyzed as mentioned before at the indicated time points post inoculation with rB95.8 (black) or rB95.8-UV (white). The amount of GFP+ cells is shown after subtraction of the background signal obtained from mock-inoculated cells. Data is represented as Mean ± SEM of 3 independent inoculations. C) *EBER1* and D) *BZLF1* gene expression was determined at the indicated time points post inoculation with rB95.8 (black) or rB95.8-UV (white) as mentioned above in HEK293 cells by RT-qPCR relative to *HMBS*. Data is represented as Mean ± SEM of 3 independent inoculations; n.d. = not detected; * = p<0.05 (unpaired t test; Holm-Sidak method).

As an initial set of experiments we performed inoculation studies to determine the optimal point in time to analyze the EBV infection in epithelial cells and to prevent an overestimation of the infection due to carryover of GFP protein and EBV RNA by the virus particles [57] and by exosomes that might be present in the virus preparation [58] We used a recombinant, prototypic EBV strain, rB95.8, that carries a green fluorescent protein (GFP), allowing the identification of infected cells by fluorescence [45]. We inoculated HEK293 cells using a spinoculation protocol [54] at a multiplicity of infection (MOI; given as infectious units/target cell) of 0.5. The frequencies GFP positive (GFP+) cells were determined by flow cytometry and EBV gene expression of the non-coding RNA *EBER1* and of the immediate-early lytic gene *BZLF1* by RT-RT-qPCR at 1, 24, 48 and 72 hours post inoculation (Fig 2). UV-inactivated rB95.8 (rB95.8-UV) served as control.

One hour post inoculation we detected a shift of the whole cell population towards a higher GFP fluorescence intensity in cells inoculated with rB95.8 and rB95.8-UV (Fig 2A; upper panel; 1 hpi), which resulted in 5.14% ± 1.31 and 5.15% ± 1.55 GFP+ cells, respectively. This amount of GFP+ cells likely reflects binding of viral particles to the cell surface and therefore carryover of GFP, especially by the UV-inactivated virus. Importantly, this shift was not longer detected at 48 hpi and 72 hpi (Fig 2A; middle and lower panel, respectively), when we detected 0.37% ± 0.08 and 0.45% ± 0.05 GFP+ cells, respectively in cells inoculated with rB95.8, whereas cells inoculated with the UV-inactivated virus showed amounts of GFP+ cells at most around background levels (0.10% ± 0.05 and 0.16% ± 0.08; Fig 2A; middle and lower panel, respectively). Fig 2B shows the compiled results for the infection frequencies after subtraction of the background signals obtained from mock-inoculated samples. Cells that were inoculated with rB95.8 showed reduced but stable amounts around 0.5% of GFP+ cells from 24 hpi to 72 hpi. By contrast, cells inoculated with rB95.8-UV showed almost no GFP+ cells (< 0.2%) within this early phase after the inoculation and which corresponds to carryover of GFP. Thus, these results demonstrate that there is a transfer of GFP and EBV RNA by the virus particles to the target cells but the majority of GFP+ cells 72 hpi is due to *de novo* synthesis of GFP. Similarly, we detected *EBER1* RNA and *BZLF1* mRNA 1 hpi with rB95.8 or with UV-inactivated rB95.8 (Fig 2C and 2D, respectively). *EBER1* expression reached the lowest level at 24 hpi, was subsequently showing an increasing trend and reached initial levels at 72 hpi when cells were inoculated with rB95.8. In rB95.8-UV-inoculated cells, *EBER1* levels were initially similar (1 hpi; p > 0.05) to those in rB95.8-inoculated cells and then they decreased to the lowest levels at 48 hpi but remained unchanged (p > 0.05) from 24 hpi to 72 hpi (Fig 2C). Similar results were obtained for *BZLF1*. We could detect *BZLF1* expression at 1 hpi both in cells inoculated with rB95.8 or with rB95.8-UV, although it was initially lower in rB95.8-UV-inoculated cells (Fig 2D). While *BZLF1* levels decreased between 24 and 72 hpi in rB95.8-inoculated cells, we could not detect *BZLF1* expression in cells that were inoculated with rB95.8-UV between 24 and 72 hpi (Fig 2D). Notably, RNA samples were subjected to DNase treatment to remove any contaminating DNA. We cannot, however, exclude carryover of transcripts by virions, which can contain various EBV transcripts [59], and exosomes that might co-precipitate during concentration of EBV by centrifugation and may contain EBERs [58]. Nevertheless, our results above indicate that carryover RNA is quickly degraded and can be distinguished from *de novo* gene expression between 24 and 72 hpi.

In summary, we conclude that carryover of GFP and EBV RNA occurs but can be distinguished from *de novo* synthesis and expression at 72 hpi. Therefore, we determined 72 hpi as optimal time point for further analyses of subsequent inoculation experiments.

### Increased hTERT expression and activity positively correlates with the infection of AGS cells by EBV

**Fig 3:**
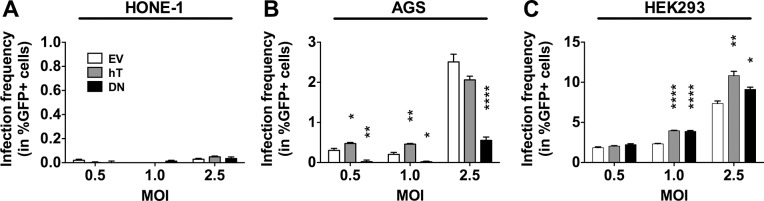
Telomerase dependent EBV infection in stably transfected epithelial cell lines. The amount of infected (in %GFP+ cells) HONE-1 (A), AGS (B) and HEK293 (C) cell lines was determined within the living cell (7-AAD negative) population by flow cytometry 3 dpi with rB95.8 after subtraction of the background signal obtained from mock-inoculated cells. Data is represented as Mean ± SEM of 3 independent inoculations. Empty vector (EV) control cells = white; hTERT (hT) overexpression = grey; DNhTERT (DN) overexpression = black; * = p<0.05; ** = p<0.01; *** = p<0.001; **** = p<0.0001 (ordinary one-way ANOVA; Dunnett’s multiple comparison test).

After establishment of cell lines stably overexpressing hTERT or DNhTERT and definition of the optimal time point for the analysis, we investigated the impact of hTERT expression and telomerase activity on the infection of these epithelial model cell lines. We used the pool of stably transfected cells and did not select for single cell clones for our experiments. As mentioned above, we inoculated the epithelial cell lines at varying multiplicities of infection (MOI; given as infectious units/target cell) and determined the frequencies of GFP positive (GFP+) cells 3 days post inoculation (dpi) by flow cytometry as shown in Fig 3.

There were almost no GFP+ cells (<0.05%) in any of the three stably transfected HONE-1 (EV, hT, or DN) cell lines 3 dpi with EBV (Fig 3A). Thus, since HONE-1-hT do not display increased hTERT activity compared to HONE-1-EV (Fig 1D) a potential positive effect of increased telomerase activity on infection frequency could not be tested here, nor could a potential negative effect of decreased telomerase activity in HONE-1 cells be assessed, since the infection frequencies were too low for HONE-1-EV. By contrast, AGS-hT cells showed significantly increased frequencies of GFP+ cells compared to AGS-EV control cells using MOIs of 0.5 and 1.0 (0.47% ± 0.05 vs. 0.30% ± 0.01; p < 0.05, and 0.46% ± 0.03 vs. 0.20% ± 0.09; p < 0.01, respectively; Fig 3B). Using MOI 2.5, AGS-hT cells showed a similar frequency of GFP+ cells compared to AGS-EV cells (2.06% ± 0.16 vs. 2.51% ± 0.33; p > 0.05; Fig 3B), suggesting that the effect of telomerase activity in increasing the frequency of cell infection with EBV can be overcome by increased numbers of virus particles per cell. The expression of the dominant negative hTERT mutant and, therefore, the suppression of telomerase activity in AGS-DN cells correlated with an almost complete absence of infection at MOIs 0.5 (0.02% ± 0.07; p < 0.01) and 1.0 (0.02% ± 0.03; p < 0.05) and with reduction of GFP+ cells at MOI 2.5 compared to in EV control cells (0.55% ± 0.14 vs. 2.51% ± 0.33; p<0.0001; Fig 3D). These results indicated that the infection of AGS cells by EBV at low MOIs is dependent on telomerase activity and suggest a contribution of telomerase activity to increased susceptibility to infection by EBV in these cells.

The frequencies of GFP+ cells in HEK293-EV, HEK293-hT and HEK293-DN cells 3 dpi with EBV using MOI 0.5 were similar (1.85% ± 0.2, 2.05% ± 0.11 and 2.23% ± 0.19, respectively). This was expected for HEK293-EV and HEK293-hT cells since these cells displayed similar telomerase activity but it was unexpected for HEK293-DN cells that had clearly lower telomerase activity (Fig 1D). Upon EBV inoculation with MOIs 1.0 and 2.5, the frequencies of GFP+ cells in HEK293-hT cells were increased compared to the EV control cells (3.96% ± 0.09 vs. 2.32% ± 0.12; p < 0.0001 and 10.84% ± 0.93 vs. 7.36% ± 0.54; p < 0.05, respectively). Again unexpectedly, we observed an increase in the frequencies of GFP+ cells in HEK293-DN cells compared to HEK293-EV control cells using MOIs 1.0 and 2.5 (3.89% ± 0.19 vs. 2.32% ± 0.12; p < 0.001 and 9.11% ± 0.49 vs. 7.36% ± 0.54; p < 0.05). These results suggested that reduction of telomerase activity in HEK293 cells that are not close to the physiological epithelium for EBV, has opposite effects with respect to EBV infection as in AGS cells that reflect more closely the physiological conditions for EBV infection.

Taken together, we observed a positive correlation between EBV infection and hTERT expression and telomerase activity in AGS cells. In HEK293 cells, however, overexpression of DNhTERT resulted in increased frequencies of GFP+ cells after inoculation with EBV, indicating that telomerase activity-associated positive effects related to EBV infection of epithelial cells might be epithelial cell background-specific and therefore the choice of the model system heavily influences the outcome of the studies.

### Increased telomerase activity associates with enhanced EBV gene expression

**Fig 4:**
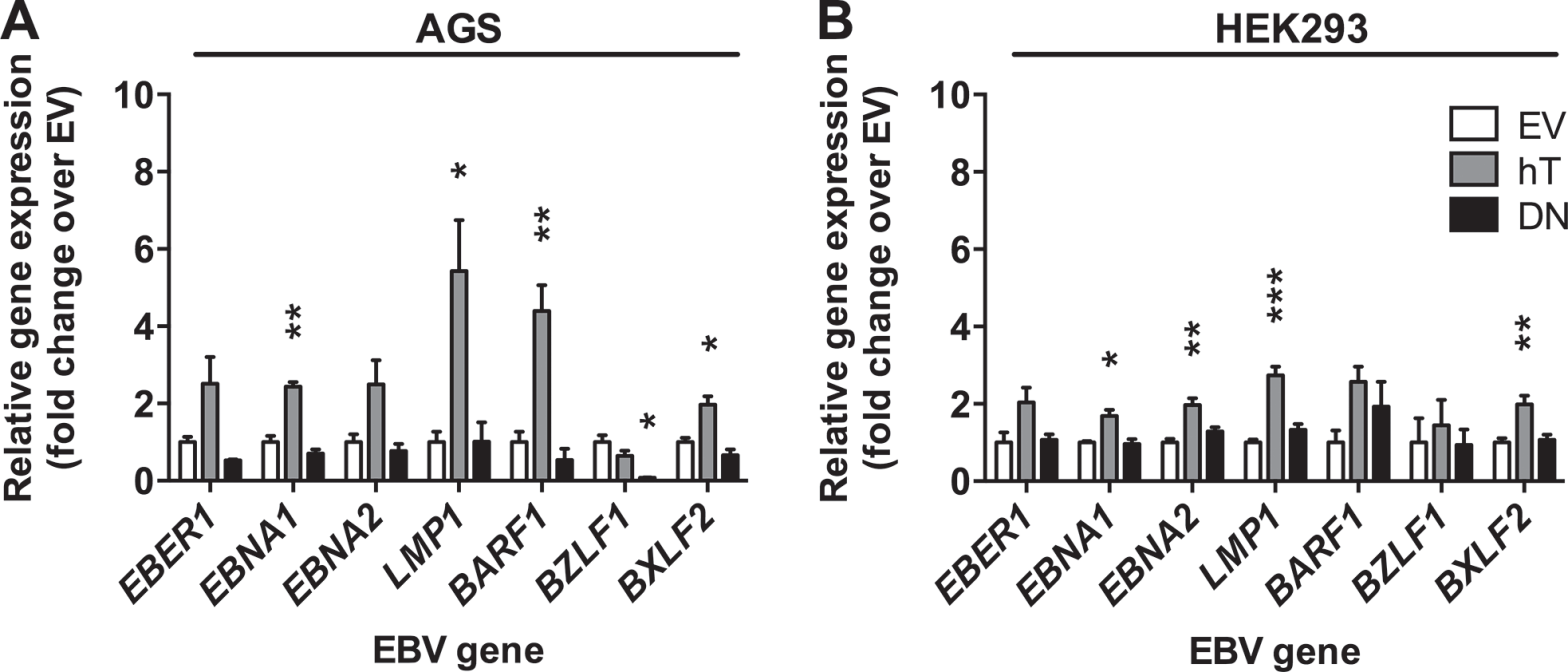
Telomerase dependent EBV gene expression in epithelial cells upon infection with rB95.8. EBV gene expression was determined in AGS (A) and HEK293 (B) empty vector control (EV; white), in hTERT (hT; grey) and in dominant negative hTERT (DN; black) cells, respectively, 3 dpi with rB95.8 at MOI 2.5 (infectious units/target cell) by RT-qPCR relative to *HMBS* and shown as fold change over EV control. Data is represented as Mean ± SEM of 3 independent inoculations; * = p<0.05; ** = p<0.01; *** = p<0.001 (ordinary one-way ANOVA; Dunnett’s multiple comparison test).

Given that it was not possible to assess telomerase activity-dependent effects on EBV infection in HONE-1 cells we focused our further experiments on AGS and HEK293 cells. Since upon inoculation with rB95.8, the expression of GFP is driven by the constitutive CMV promoter [45], GFP expression is not completely identical with infection of the cells resulting in EBV gene expression or replication. Therefore, we determined EBV gene expression of the non-coding RNA *EBER1* and *BZLF1* as mentioned above and additionally of three latency-associated genes *EBNA1*, *EBNA2*, and *LMP1*, of *BARF1* and of the late lytic gene *BXLF2* in AGS cells and HEK293 cells upon inoculation at MOI 2.5 (Fig 4A and 4B), as these cells had demonstrated the highest frequencies of GFP+ cells 3 dpi with EBV using this MOI (Fig 3B and 3C). We detected expression of all EBV genes tested in our panel and of *EBER1* in all stably transfected AGS and HEK293 cell lines (Fig 4). The most abundant transcripts were *EBNA1* in AGS cells and *EBER1* in HEK293 cells (data not shown). Increased hTERT expression and telomerase activity in AGS-hT cells correlated with increased transcription of all EBV genes including *EBER1* with the exception of *BZLF1* that was reduced to 0.7-fold, compared to EV control cells (Fig 4A). The strongest increases in transcription in AGS-hT cells compared to AGS-EV control cells were recorded for *LMP1* (5.4-fold), *BARF1* (4.4-fold), *EBNA1* (2.4-fold), and *BXLF2* (2.0-fold). Conversely, suppression of telomerase activity in AGS-DN cells resulted in lower or similar (*LMP1*) expression of all tested genes compared to EV control cells. Thereby, *BZLF1* expression (0.1-fold) showed significant reduction in AGS-DN in comparison to AGS-EV cells (Fig 4A). The overexpression of hTERT in HEK293-hT cells led as well to the up-regulation of all EBV genes tested and *LMP1* showed the strongest and most significant up-regulation of 2.7-fold over EV control cells (Fig 4B). Similarly, expression of *EBNA1* (1.7-fold), *EBNA2* (2.0-fold) and *BXLF2* (2.0-fold) were significantly higher in HEK293-hT cells compared to HEK293-EV cells (Fig 4B). EBV gene expression in HEK293-DN cells was comparable to in HEK293-EV control cells (Fig 4B), although HEK293-DN cells exhibited reduced telomerase activity compared to HEK293-EV cells (Fig 1D).

Taken together, these results indicate that the expression of EBV genes is influenced by telomerase activity in both AGS and HEK293 cells, since the increase and the reduction of telomerase activity resulted in up-regulation and down-regulation of EBV gene expression, respectively. This, in turn, also suggests that the differences observed between AGS cells and HEK293 cells with respect to frequencies of GFP+ cells related to telomerase activities after inoculation with EBV must not be mirrored in differences of EBV gene expression.

### Increased infection frequencies of AGS-hT and decreased infection frequencies of AGS-DN cells are observed with distinct EBV strains

To confirm and expand our observation that hTERT overexpression associates with increased infection frequencies, while expression of DNhTERT results in reduced infection frequencies in AGS cells, we tested two additional EBV strains on the AGS model cell lines that also reflect are more physiological background for an infection by EBV as mentioned above. Next to rB95.8, we employed the EBV strain M81 (rM81), which was originally isolated from a Chinese patient with NPC and that shows an increased epithelial cell tropism as compared to the prototypic EBV strain B95.8 [44]. The other additional EBV strain was a B95.8-based EBV with a *BZLF1*-knockout (rBZLF1-KO) that is not able to undergo lytic replication [46]. The amount of GFP+ cells was again determined 3 dpi with EBV by flow cytometry.

**Fig 5:**
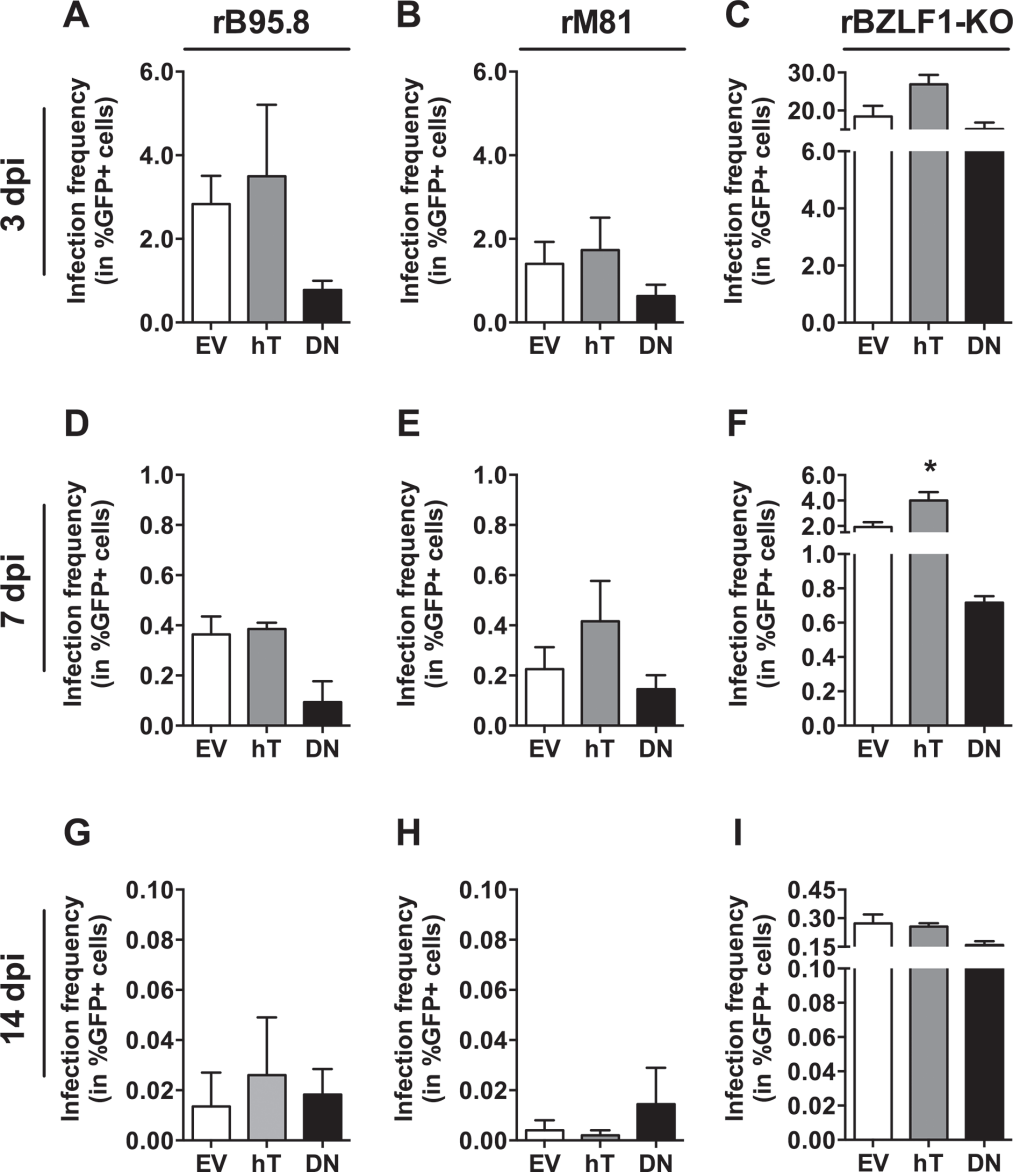
Infection frequencies of AGS cell lines upon infection with different EBV strains. The amount of infected (%GFP+) AGS-EV (empty vector control; white), AGS-hT (hTERT overexpression; grey) and AGS-DN (DNhTERT overexpression; black) cells was determined within the living cell (7-AAD negative) population by flow cytometry 3 (A-C), 7 (D-F) and 14 (G-I) dpi with rB95.8 (A, D, G), rM81 (B, E, H) and rBZLF1-KO (C, F, I) after subtraction of the background signal obtained from mock-inoculated cells. Data is represented as Mean ± SEM of 3 independent inoculations.

The control inoculation with the prototypic EBV strain rB95.8 (Fig 5A) showed similar results compared to previous experiments (Fig 3B). Slightly increased, although not significant, frequencies of GFP+ cells were detected in AGS-hT (3.50% ± 1.71) as compared to AGS-EV cells (2.83% ± 0.68), whereas AGS-DN cells showed a reduced amount of GFP+ cells (0.78% ± 0.22) compared to AGS-EV.

Given its increased tropism for epithelial cells, we expected higher frequencies of GFP+ cells with rM81 EBV. In general, we detected somewhat lower amounts of GFP+ cells (Fig 5B) as we had seen after inoculation with rB95.8, however, the reasons are not clear. Nevertheless, the pattern was similar, with higher frequencies of GFP+ cells in AGS-hT (1.73% ± 0.77) and decreased amounts of GFP+ cells in AGS-DN (0.63% ± 0.27) as compared to AGS-EV control cells (1.40% ± 0.52) (Fig 5B). Interestingly, the inoculation with the rBZLF1-KO virus resulted overall in about 10-fold higher amounts of GFP+ cells (Fig 5C), again with the same tendency that AGS-hT showed increased amounts of GFP+ cells (26.90% ± 2.45) and AGS-DN lower frequencies of GFP+ cells (15.13% ± 1.70) as compared to AGS-EV cells (18.43% ± 2.78).

**Fig 6:**
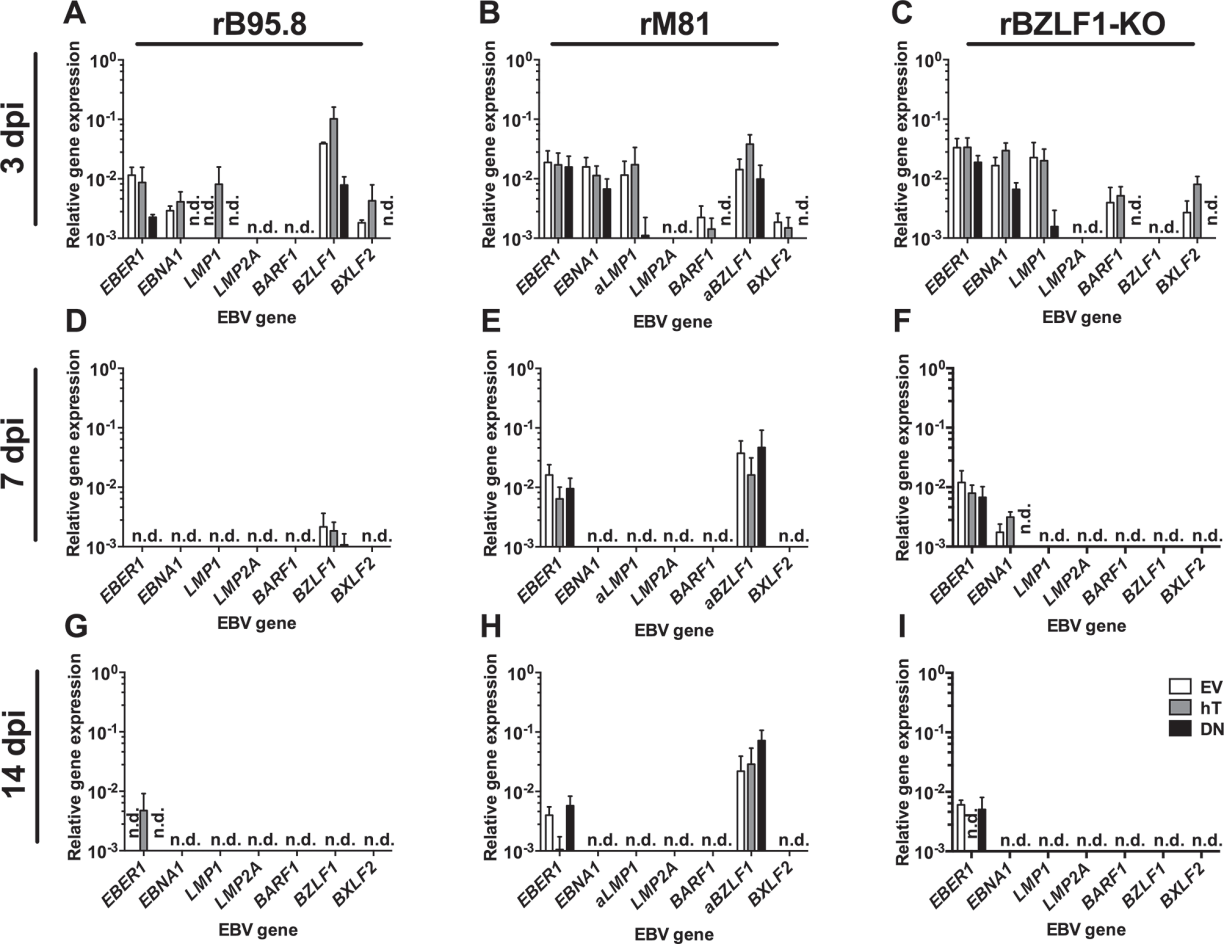
EBV gene expression in AGS cell lines upon inoculation with rB95.8, rM81 and rBZLF1-KO. EBV gene expression was determined in AGS empty vector control (EV; white), in hTERT (hT; grey) and in dominant negative hTERT (DN; black) cells, respectively, 3 (A-C), 7 (D-F) and 14 dpi (G-I) with rB95.8 (A, D, G), rM81 (B, E, H) and rBZLF1-KO (C, F, I) by RT-qPCR relative to *HMBS*. Data is represented as Mean ± SEM of 3 independent inoculations; n.d. = not detected.

Taken together, inoculations with all three virus strains resulted in increased amounts of GFP+ AGS-hT cells and decreased frequencies of GFP+ AGS-DN cells as compared to the amount of GFP+ cells in AGS-EV. The much higher frequencies of GFP+ cells obtained with the rBZLF1-KO virus could be explained by the fact that there is no *BZLF1* mRNA expression contrasting the rB95.8 and the rM81 strains (Fig 6A–6C: 3dpi), suggesting lytic replication in the latter and loss of infected cells through subsequent lysis.

**Fig 7:**
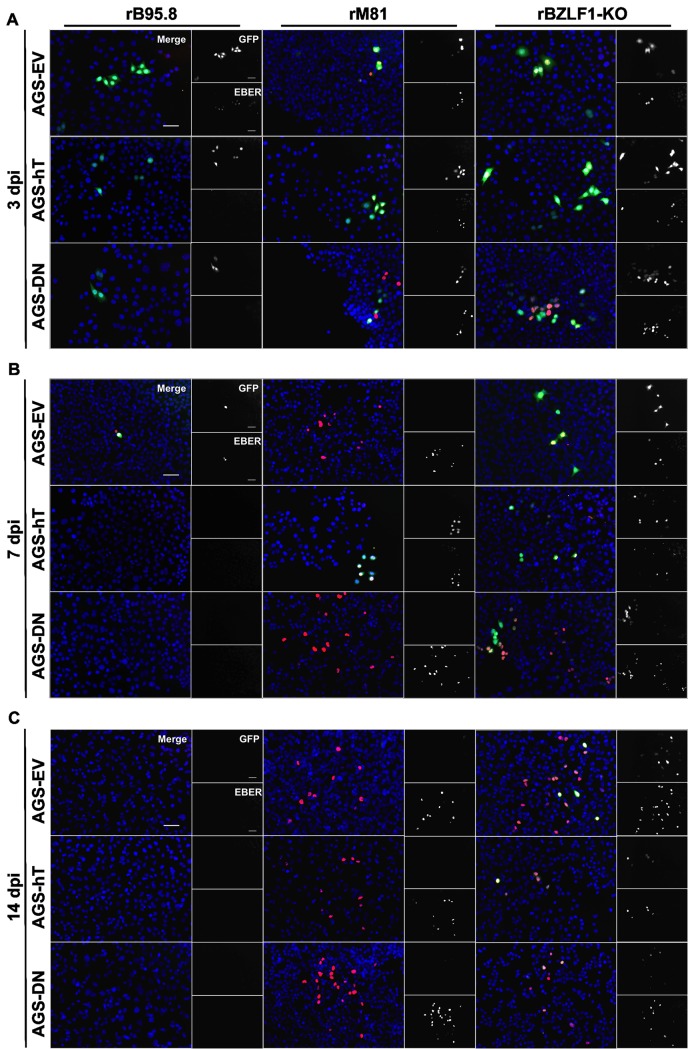
Detection of EBV-infected AGS cells by EBER-FISH upon infection with rB95.8, rM81 and rBZLF1-KO. Infection of AGS-EV (empty vector control), AGS-hT (hTERT-overexpression) and AGS-DN (DNhTERT-overexpression) cells was confirmed by EBER-FISH 3 (A), 7 (B) and 14 (C) dpi. Nuclei are stained with DAPI (blue). GFP expressing cells appear green, EBER expressing cells appear red and double positive cells appear with yellow nuclei in merged pictures. Pictures shown are representative for three independent experiments; Scale bar = 50 μm.

To further verify EBV infection in these cells we performed fluorescence *in situ* hybridization (FISH) experiments to detect *EBER* expressing cells 3 dpi as shown in Fig 6A. Infected epithelial cells might actually lose GFP expression, which would lead to an underestimation of the infection, especially upon inoculation with rM81. Surprisingly, at 3 dpi with rB95.8 we found only GFP+ cells and cells without or at most with very low *EBER* expression (Fig 7A; left panel). In contrast, at 3 dpi with rM81 (Fig 7; middle panel) and rBZLF1-KO virus, (Fig 7; right panel), respectively, almost all infected cells appeared to be double positive for GFP and *EBER1* (GFP+/*EBER*+). Notably, especially cells with strong GFP expression were clearly *EBER* positive, indicating that either more virus per cell entered or that GFP expression from this recombinant virus is more efficient since it does not undergo lytic replication which would lead to loss of infected cells by cell lysis. When AGS-DN cells were inoculated with rM81 or rBZLF1-KO virus about half of the infected cells were positive only for *EBER* and did not show any GFP expression or only very low (Fig 7; lower middle and lower right panel). This suggested additionally that hTERT might influence GFP expression from the CMV promoter of these recombinant viruses. In summary, we might actually overestimate the infection frequencies as determined by flow cytometry for the infections with rB95.8 and underestimate the infection frequencies for the other two viruses.

Next, we determined EBV gene expression as described above to confirm an ongoing infection within the cells 3 dpi. Notably, we did DNAse treatment of the samples to prevent detection of contaminating DNA including unspliced *BZLF1* DNA. Moreover, we ascertained that *LMP2A* mRNA from rM81 virus is detected very well by our RT-qPCR assay. Overall EBV gene expression levels were lower (Fig 6A–6C) compared to the previous experiments (Fig 4). Nevertheless, 3 dpi with EBV we detected transcription of all tested genes upon inoculation with rB95.8 except *LMP2A* and *BARF1* (Fig 6A), while we could not detect *LMP2A* expression upon inoculation with rM81 (Fig 6B) and, as expected, no *BZLF1* expression upon inoculation with rBZLF1-KO (Fig 6C). Upon inoculation with rB95.8, expression of the latency genes *EBNA1* and *LMP1* and the lytic genes *BZLF1* and *BXLF2* was increased in AGS-hT cells compared to AGS-EV control cells (Fig 6A). At the same time, expression of *EBER1* and *EBNA1* was reduced and of *BXLF2* absent in AGS-DN cells upon inoculation with rB95.8 (Fig 6A). Expression of *LMP2A* and *BARF1* was not present in all three cell lines (Fig 6A). AGS-hT and AGS-DN cells inoculated with rM81 showed reduced or absent expression of all genes tested, except for *BZLF1* that showed increased expression compared to AGS-EV cells (Fig 6B). AGS-hT cells inoculated with rBZLF1-KO showed increased expression of *EBNA1*, *BARF1* and interestingly *BXLF2*, while the expression of all EBV genes tested was reduced or even absent in AGS-DN cells compared to AGS-EV cells (Fig 6C). Expression of *BXLF2* in the absence of *BZLF1* indicates that *BXLF2* expression might be regulated by other factors, e.g. *BRLF1* or deltaNp63, as is the case for BARF1 [60,61].

In summary, the trend of an increased, telomerase-related EBV gene expression in AGS was confirmed at 3 dpi using two additional EBV strains.

### Loss of GFP expression does not necessarily correlate with loss of EBV

To confirm that increased hTERT expression and telomerase activity in AGS cells can contribute to the establishment of the EBV infection and assess if they support EBV maintenance within these epithelial cells, the infection was followed for additional 14 days by flow cytometry (Fig 5D–5I), *EBER*-FISH (Fig 7B and 7C), RT-qPCR (Fig 6D–6I).

Table 1 summarizes the main findings from Figs 5–7. Following inoculation with rB95.8 GFP expression was lost very rapidly, dropping below 0.5% GFP+ cells at 7 dpi and below 0.06% at 14 dpi (Fig 5D and 5G). Similar results were obtained following inoculation with rM81 (Fig 5E and 5H) although higher numbers of GFP+ AGS-hT cells (0.42% ± 0.16) were detected 7 dpi compared to AGS-EV cells (0.23% ± 0.09) (Fig 5E). Cells inoculated with rBZLF1-KO (Fig 5F and 5I) showed the highest frequencies of GFP+ cells in AGS-hT (4.01% ± 0.66; p = 0.0271) that were significantly increased 7 dpi compared to EV-control cells (1.91% ± 0.39) (Fig 5F). However, the amount of GFP+ cells following inoculation with rBZLF1-KO dropped as well below 0.4% 14 dpi (Fig 5I). Interestingly, AGS-DN cells showed still the lowest frequencies of GFP+ cells at 7 dpi compared to EV-control cells (Fig 5D–5F), while 14 dpi this was only the case for rBZLF1-KO inoculated AGS-DN cells (Fig 5I).

**Table 1:** Summary of main findings from Figs 5–7.

Figure	Factor analyzed	Days post inoculation	EBV strain		
			rB95.8	rM81	rB95.8-BZLF1-KO
**5**	**Frequency of GFP+ cells**	**3**	**++**	**++**	**+++**
		**7**	**+**	**+**	**++**
		**14**	**-**	**-**	**+**
**6**	**GFP / EBER expression**	**3**	**+ / (+)**	**+ / +**	**+ / +**
		**7**	**(+) / -**	**(+) / +**	**+ / +**
		**14**	**- / -**	**- / +**	**(+) / +**
**7**	**Gene expression**	**3**	**+ (*EBER1*, *EBNA1*, *LMP1*, *BZLF1*, *BXLF2*)**	**+ (*EBER1*, *EBNA1*, *LMP1*, *BARF1*, *BZLF1*, *BXLF2*)**	**+ (*EBER1*, *EBNA1*, *LMP1*, *BARF1*, *BXLF2*)**
		**7**	**(+) (*BZLF1*)**	**+ (*EBER1*, *BZLF1*)**	**+ (*EBER1*, *EBNA1*)**
		**14**	**(+)(*EBER1*)**	**+ (*EBER1*, *BZLF1*)**	**(+) (*EBER1*)**

- = no expression or not detected; (+) = barely expressed or not in all cell lines detected; + = expressed.

*EBER*-FISH experiments 7 dpi and 14 dpi (Fig 7B and 7C) revealed that in cells inoculated with rB95.8 almost no GFP+ and/or *EBER*+ cells were detected 7 dpi except of very few cells in AGS-EV (Fig 7B, upper left panel) and that rB95.8 or at least GFP and *EBER* expression was completely lost 14 dpi (Fig 7C; left panel). In contrast, *EBER*+ cells were detected in all stably transfected AGS cell lines inoculated with rM81 (Fig 7B, middle panel) but GFP+ cells were detected only in AGS-hT 7 dpi (Fig 7B; middle panel). GFP expression was as well completely lost 14 dpi upon infection with rM81 (Fig 7C; middle panel). As expected from the flow cytometry data, GFP+ cells were detected in *EBER*-FISH assays 7 dpi and 14 dpi with rBZLF1-KO (Fig 7B and 7C; right panel). However, also cells that were either GFP+ or *EBER*+ were detected. Especially for AGS-DN cells 7 dpi more *EBER*+ then GFP+ or double positive cells were detected (Fig 7B; lower right panel). As shown for the inoculations with rM81, mostly *EBER*+ and only few double positive cells were detected 14 dpi with rBZLF1-KO (Fig 7C; right panel).

Similar to the flow cytometry data in Fig 5 we detected decreasing EBV gene expression levels over time (Fig 6). When cells were infected with rB95.8, almost no expression of EBV genes was detected 7 and 14 dpi (Fig 6D and 6G). Only *LMP1* in AGS-hT, *BZLF1* in all AGS cell lines was detected 7 dpi (Fig 6D), while expression of *EBER1* was detected in AGS-hT cells 14 dpi (Fig 6G). All remaining tested genes were not detected or barely reached the limit of detection. Cells infected with rM81, showed reduced expression of *EBER1*, *EBNA1* and *BXLF2* 7 dpi, while *BARF1* expression was lost completely and *LMP1* as well as *LMP2A* were not detected (Fig 6E). The expression of *EBER1* was further reduced 14 dpi with rM81 and *EBNA1* and *BXLF2* were not detected anymore (Fig 6H). Interestingly, *BZLF1* expression was detected in rM81-inoculated cells at all three time points with more or less unchanged levels (Fig 6B, 6E and 6H). In rBZLF1-KO-inoculated cells the expression of EBV genes was also lost or reduced over time (Fig 6C, 6F and 6I). While expression of *EBER1* in all three AGS cell lines and *EBNA1* in AGS-EV and AGS-hT was still detected 7 dpi (Fig 6F), only *EBER1* could be detected weakly at 14 dpi (Fig 6I). The expression levels of the remaining EBV genes, tested in our panel, barely reached the limit of detection or were not detected at all.

Taken together, these results indicate that determining infection frequencies by flow cytometry on the basis of GFP expression gives a good correlation for short-term infections (3 dpi) with the three virus strains tested, but might become unreliable especially for rM81 and rBZLF1-KO as shown by *EBER*-FISH. However, because of the more qualitative nature of the *EBER*-FISH assays and the low EBV gene expression levels we can only confirm a contribution of hTERT expression and telomerase activity to the establishment of an EBV infection 3 dpi. Nevertheless, these results indicated that lymphotropic EBV strains such as B95.8 can establish an infection and be maintained in AGS cells only when lytic replication is impaired, while EBV strains with increased epithelial tropism like M81 can readily do so.

## Discussion

In this study we investigate the impact of hTERT expression and telomerase activity on the infection frequency of epithelial cells by EBV *in vitro* and how infection develops over time. We found that increased telomerase activity contributes to enhancing EBV infection of AGS cells within 3 dpi by generating an environment that facilitates EBV’s gene expression in cells overexpressing hTERT. Moreover, we found that infection frequency of AGS cells by EBV was influenced by telomerase activity in a distinct way compared to HEK293 cells, suggesting a decisive role of the cellular background. On the other hand, in HONE-1 cells the high endogenous hTERT levels proved cells to become largely refractory to EBV. Our results indicate that infection of epithelial cells by EBV is a very selective process involving among others telomerase activity and cellular background.

Our observation that increased telomerase activity associates with enhanced EBV gene expression in AGS cells in the first 72 hours after inoculation with the virus is unprecedented. Importantly, we observed enhanced EBV gene expression in AGS-hT cells and conversely reduced expression in AGS-DN cells using three EBV strains including a lymphotropic, an epitheliotropic and a replication incompetent strain. Thus, experiments on hTERT gain and loss of function clearly documented the functional link between telomerase activity and the magnitude of EBV genes expression in AGS cells using as well distinct EBV strains (Figs 4 and 7; 3 dpi). Moreover, we observed an augmented infection frequency with lower MOIs in AGS-hT cells. Thus, increased telomerase activity enhances the frequency of AGS cell infection with EBV that becomes detectable after exposure to low virus titers compared to cells with markedly less telomerase activity. These results suggest a contribution of telomerase activity in AGS cells to either increased susceptibility to infection *via* factors influencing binding, entry, nuclear translocation, circularization, DNA replication, assembly or maturation.

A remarkable finding was that despite detection of latent EBV gene expression in the first 72 hours following EBV inoculation latency was not established in AGS cells. We detected expression of *EBNA2*, i.e. the latent master regulatory EBV gene [62–65], *EBNA1* the latent EBV gene responsible for mitotic segregation and maintenance of the virus episome [66–69], and the oncogenic latent EBV gene *LMP1* plus the epithelial-specific latent *BARF1*. Nevertheless, we did not detect expression of *LMP2A*, suggesting that *EBNA2* expression did not ignite *LMP2A* expression as observed in B cells [62,63]. Our findings are in line with those of Shannon-Lowe *et al*. [19] who compared EBV gene expression in epithelial cells to that in B cells after *in vitro* EBV inoculation. They found that only a minority of EBV-infected epithelial cells, as documented by *EBER* detection, expressed EBNA1 and that EBNA1 in epithelial cells exclusively originated from Qp promoter contrasting to EBNA1 in newly infected B cells that originated from Cp/Wp promoters. Our results also confirm that EBV is lost from epithelial cells during prolonged culture [19]. Our data suggest that, although EBV is capable to bind and enter into AGS cells and even induce expression of important latency-associated viral genes, the establishment of stable viral persistence in AGS is not achieved, which may be due to the lack of appropriate adapter molecules to link EBV gene products into host signaling pathways, as occurs in B-cells.

The detection of the master regulatory lytic gene *BZLF1* and of the late lytic gene *BXLF2* suggested induction and completion of the viral lytic cycle in EBV inoculated AGS cells. Several investigators have reported expression of *BZLF1* [1,2,19,47,70–74] but not expression of *BXLF2*. Our findings on lytic EBV gene expression in AGS cells contrast those in B cells with respect to hTERT expression. Indeed, hTERT silencing leads to increased *BZLF1* gene expression and hTERT expression inhibits lytic EBV replication in B cells [40,41]. Despite detectable expression of *BXLF2* in AGS cells, we could not detect EBV particle production. The likely reason for this might be that since the maximal infection frequency of both replication-competent EBV strains used here ranged between 2% to 5%, newly formed EBV particles might have escaped detection. An alternative explanation might be that the majority of virus is undergoing an abortive replication, as it was reported to occur by Strong and colleagues [75]. Notably, replication of EBV in epithelial cells, however, seems to be important for host-to-host transmission of the virus as epithelial cell-derived EBV is more lymphotropic than B cell derived [11]. Furthermore, there is some evidence that oropharyngeal epithelium may act as an amplifier for EBV shed into saliva [5]. Thus, EBV seems to have evolved to exploit B cells as reservoir within the host and utilizes only selected epithelial cells to facilitate host-to-host transmission.

Inoculation of stably transfected HEK293 cells with EBV revealed a quite discordant infection pattern in relation to telomerase activity compared to that observed in stably transfected AGS cells. We observed higher expression of *BZLF1* HEK293-DN compared to in HEK-293-EV cells, thus contrasting the findings in the corresponding AGS cells. Shannon-Lowe and colleagues also noted distinct EBV gene expression in primary epithelial cells, AdAH cells, and AGS cells [19]. HEK293 cells originate from kidney epithelium, suggesting that telomerase activity may affect EBV infection in an opposite way if they originate from an anatomical location remote from the portal of entry and exit of EBV. Since HONE-1 cells originate from the oropharynx one might expect them to exhibit a similar behavior to AGS cells. We could not increase telomerase activity in HONE-1 cells most likely due to the relatively high telomerase basal activity compared to in AGS cells. Nevertheless, infection of HONE-1 cells was not possible in our experimental setup. Since HONE-1 lost EBV *in vitro* [20] the lack of infection after inoculation with EBV here despite the relatively high baseline telomerase activity may suggest that the cells are resistant to infection by free EBV particles. Interestingly, Tsang and colleagues [24] showed infection of HONE-1 cells by cell-to-cell contact with lytically induced EBV positive BL cells, indicating for HONE-1 cells a distinct mode of infection.

A limitation of our study is that we used transformed cells and not primary epithelial cells. The vast majority of cancer cell lines have to some extent an increased telomerase activity, which is a hallmark of cancer [76,77]. This is also the case for our epithelial model cell lines, since hTERT was already endogenously expressed and telomerase activity was detectable. However, we are convinced that at least the cell line AGS is suitable to study the impact of telomerase activity on the infection of epithelial cells since they have a relatively low endogenous telomerase activity compared to the other epithelial cell lines used here. Interestingly, this is reflected by immunohistochemical studies on oro-nasopharyngeal and tonsillar tissues, which demonstrated that replicating EBV can only rarely be detected in epithelial cells, except in the case of oral hairy leukoplakia [1,2,78,79].

Our data from the AGS cells indicate that telomerase activity is critical for enhancing EBV latent genes. *In vivo* this would be relevant only in basal cells of nasopharyngeal and gastric epithelia since telomerase activity is mostly restricted to these cells. It is possible that in *vivo* EBV infection and expression of latent genes in the basal cells could be substantially increased by telomerase activity. This may play a critical role in initiation of EBV-associated neoplastic processes in the nasopharyngeal and gastric epithelia. Indeed, EBV infection of basal cells was hypothesized recently [80]. Our findings contribute to this notion that EBV might infect basal epithelial cells with increased telomerase activity, probably *via* cell-to-cell contact. These epithelial cells are then able to support a short-lived infection by EBV. Subsequent differentiation of infected basal epithelial cells could then potentially facilitate lytic EBV replication. Such replication might not be so critical for the development of malignancy but may be important in production of progeny virus that is shed into saliva for host-to-host transmission. Finally, our findings may contribute to the current hypothesis that, when additional factors are involved, e.g. telomerase deregulation, allelic deletions, genetic and epigenetic alteration or dysregulated cell signaling pathways, as seen in NPC [23,30,56,81–85], EBV may be able to infect and establish an infection in epithelial cells.
